# Generation of Pure State Photon Triplets in the C-Band

**DOI:** 10.3390/mi10110775

**Published:** 2019-11-13

**Authors:** Xi-Rong Su, Yi-Wen Huang, Tong Xiang, Yuan-Hua Li, Xian-Feng Chen

**Affiliations:** 1 State Key Laboratory of Advanced Optical Communication Systems and Networks, Department of Physics and Astronomy, Shanghai Jiao Tong University, Shanghai 200240, China; suxirong@sjtu.edu.cn (X.-R.S.); 19900104@sjtu.edu.cn (T.X.); lyhua1984@163.com (Y.-H.L.); 2 Department of physics, Jiangxi Normal University, Nanchang 330022, China

**Keywords:** pure state, cascaded spontaneous parametric down-conversion (SPDC), numerical simulation

## Abstract

In this work, the cascaded second-order spontaneous parametric down-conversion (SPDC) is considered to produce pure state photon triplets in periodically poled lithium niobite (PPLN) doped with 5% MgO. A set of parameters are optimized through calculating the Schmidt number of two-photon states generated by each down-conversion process with different pump durations and crystal lengths. We use a Gaussian filter in part and obtain three photons with 100% purity in spectrum. We provide a feasible and unprecedented scheme to manipulate the spectrum purity of photon triplets in the communication band (C-band).

## 1. Introduction

The scheme of generating photon pairs using cascaded second-order spontaneous parametric down-conversion (SPDC) [1,2] is an indispensable ingredient of modern quantum technology and has great potential in many applications, such as quantum cryptography [3], quantum teleportation [4] and quantum entanglement swapping [5]. Recently, a wide variety of methods have been proposed to produce photon triplets. Common methods include direct generation of photon triplets [6,7,8], the process of four wave mixing (FWM) [9,10,11,12] and generation of three entangled photons by cascaded second-order SPDC [13,14,15,16]. Some studies propose implementing third-order SPDC in optical fibers and bulk crystals. There are always low count rates for schemes based on the χ^(3)^ process. The FWM techniques consists of stimulated SPDC and cascaded FWM. The latter can be divided into three categories according to the different ways of cascading. The cascaded second-order SPDC is considered because of the simple model, which consists of two second order SPDC processes. The mature theory and substantial experiments make it a reliable scheme.

The research about quantum correlation among individual photons lies at the core of quantum technologies. Under different conditions, the two-photon generated by SPDC will present a state of frequency positive correlation, inverse correlation or uncorrelation. The last method is used to provide a heralded source [17,18]. Previous experiments have failed to give a specific theoretical numerical analysis to judge the spectral purity of the generated photon pairs. The full use of filters [19,20] will greatly reduce the coincidence counting rate. Recently, Zhang et al. decomposed the factor mathematically to manipulate the tripartite frequency correlation [21]. But the spectrum of photons after the first SPDC and the effect on the second order down-conversion were not taken into account. They produce photons with wavelength of ~3000 nm, which is almost unavailable. So far, there is little theoretical work about pure states photon triplets in the C-band.

Quantum interference is vital for quantum information science. It is not only the basis of quantum manipulation technology, but also an important tool to implement quantum computing and quantum communication. The realization of quantum computation [22] depends on the measurement and reading of quantum states, and quantum interference is one of the most simple and feasible methods for quantum measurement. Quantum communication [23,24] is more dependent on the transmission and acquisition of information by means of interference. Three-photon interference is critical for the exploitation of quantum information in higher dimensions [25]. The GHZ interference is observed in the experiment, which lays the foundation for the subsequent quantum secret sharing [26]. In general, the photon triplets generated by the cascaded SPDC will have correction in frequency. This allows the photon pair to be resolved in the frequency dimension, thereby reducing the visibility of the interference [27]. For instance, the interference of indistinguishable photons makes the entanglement swapping and teleportation possible, which in turn opens up prospects for distributing of entanglement between distant matter qubits. The goal of our work is to prepare three photons with hyperspectral purity, which are critical for research into quantum information processes.

In this work, the suitable pump duration and crystal length are selected to eliminate the frequency correlation between the photon pairs in each SPDC process. In terms of the theoretical analysis, spectral purity of photon pairs is mainly measured by means of Schmidt number [28,29]. The conclusion of our theoretical calculation is supported by the two photons’ and three photons’ joint spectrum. Relevant theories will be discussed in Section 2. The common pump source used to acquire polarization-entangled photon pairs from SPDC is narrow-band or continuous wave (CW) laser, but the subsequent photon pairs have a strong correlation in frequency [30]. A broadband pumping source is adopted in our work, and the optimal pumping duration is chosen by numerical investigation in Section 3. Finally, we obtain pure-state photon triplets with two kinds of periodically poled crystals under different parameters.

## 2. Tripartite State and Joint Spectrum

### 2.1. Model

Quasi-phase matching is adopted because of the simpler and more flexible matching condition. The theoretical model consists of two parts, which are two nondegenerate SPDC processes [13]. A pair of photons called idler photons *ω*_0_ and signal photons *ω*_1_ are generated from the first SPDC process. The idler photons continue to be the pump source of the second SPDC process, producing photons *ω*_2_ and *ω*_3_.

**Figure 1:**
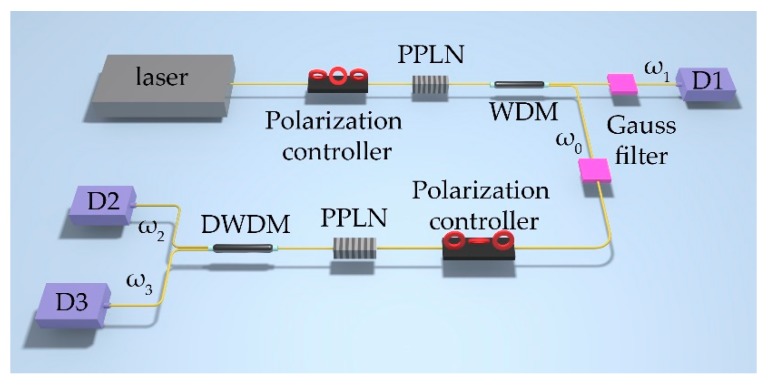
Theoretical model of cascaded second-order SPDC. Photons *ω*_0_ and *ω*_1_ are generated in the first crystal and then photons *ω*_2_ and *ω*_3_ are generated from the second crystal. The two parts of this model are periodically poled lithium niobite (PPLN) with lengths *L*_1_ and *L*_2_ respectively. Two Gauss filters are used to manipulate the joint spectrum of photonic pairs.

The phase-matching conditions of the two processes are type e → o + o and type e → e + o, respectively. As shown in Figure 1, the lengths of the two crystals are *L*_1_ and *L*_2_ while the periodicities are Ʌ_1_ and Ʌ_2_, respectively. When the pump light with center frequency of *ω*_0_ is incident into the first crystal, the generated photon pairs will be correlated in time and frequency due to the conservation of energy and momentum. The relation between the wave vectors and the frequency of the three photons are *k_p_* = *k*_1_ + *k*_0_ + *k_g_*_1_ and ℏ*ω_p_* = ℏ*ω*_1_ + ℏ*ω*_0_, where *k_g_*_1_ = 2π*m*/Ʌ_1_ is the compensated wave vector. In the second down-conversion process, photon *ω*_0_ splits into *ω*_2_ and *ω*_3_ while the conservation conditions are also satisfied, which are *k*_0_ = *k*_2_ + *k*_3_ + *k_g_*_2_ and ℏ*ω*_0_ = ℏ*ω*_2_ + ℏ*ω*_3_, where *k*_g2_ = 2π*m*/Ʌ_2_. Therefore, in the whole frequency conversion process, the energy conservation and momentum conservation are also satisfied between the initial pump photon and the resulting three photons.

### 2.2. Hamiltonian and Probability Amplitude Function

For the convenience of calculation, our model adopts a one-dimensional collinear phase-matching structure. Since the pump field is strong, the field is treated as an electric classical field *E_p_* (**r**, *t*) = $\tilde{\alpha }$(*t*) exp [*ik_p_*(*ω_p_*)*z*], rather than using the annihilation operator of the pump photon. A Gauss envelope is chosen as the pump function $\tilde{\alpha }$*_p_*(*t*) = $\tilde{\alpha }$*_p_*(0) exp(−*t*^2^/2*τ_p_*^2^). The expression corresponding to the frequency domain is

(1) $$\alpha \left(\Omega_{p}\right)=\frac{\tau_{p}}{\sqrt{2\pi }}\exp(-\frac{\tau_{p}^{2}\Omega_{p}^{2}}{2})$$

where $\tau_{p}$ is the pump duration and $\Omega_{p}=\omega_{p}-{\bar{\omega }}_{p}$ is the frequency difference.

After calculating the integral of Hamiltonian [31] and simplifying the statements, the final expression of the two-photon state is

(2) $$|\psi_{2}\rangle =\int_{t_{0}}^{t}dt^{\prime }{\hat{H}}_{I}\left(t^{\prime }\right)=A\int d\omega_{s}\int d\omega_{i}{\hat{a}}_{s}^{†}\left(\omega_{s}\right){\hat{a}}_{i}^{†}\left(\omega_{i}\right)\alpha \left(\omega_{s},\omega_{i}\right)\varphi \left(\omega_{s},\omega_{i}\right)|0\rangle +c.c.$$

where *α*(*ω_s_*, *ω_i_*) and *φ*(*ω_s_*, *ω_i_*) are pump envelope function and phase-matching function, respectively. Their product is the two-photon amplitude function

(3) $$F(\omega_{s},\omega_{i})=\alpha \left(\omega_{s}+\omega_{i}\right)\varphi \left(\omega_{s},\omega_{i}\right)$$

The phase matching function in upper equation is

(4) $$\varphi \left(\omega_{s},\omega_{i}\right)=\sin c\left[\frac{\left(k_{s}\left(\omega_{s}\right)+k_{i}\left(\omega_{i}\right)-k_{p}\left(\omega_{s}+\omega_{i}\right)+k_{g}\right)L}{2}\right]$$

For simpler operation, a coefficient *γ* = 0.193 is introduced to approximate the sinc function to a Gauss function to ensure that they have the same full width at half maximum (FWHM). This approximation only removes the small peak of sinc function and has no effect on the calculation of biphoton joint spectrum.

Assuming a perfect phase-matching condition, we carry out the Taylor expansion of the wave vector and preserve the first order term. That is $k_{m}\left(\omega_{m}\right)=k_{m0}+{k^{\prime }}_{m}\left(\omega_{m}-{\bar{\omega }}_{m}\right)+\cdots$, ${k^{\prime }}_{m}=\partial k_{m}\left(\omega \right)/\partial \omega {|}_{\omega ={\bar{\omega }}_{m}}$(*m* = *p*, *s*, *i*). The influence of group velocity dispersion and higher order terms are not considered. The second derivative of wave vector does not change obviously with the wavelength. In addition, in the actual system, the error caused by dispersion can be overcome by compensation. The phase-matching function is described by

(5) $$\varphi \left(\omega_{s},\omega_{i}\right)\approx \exp\left\{-\gamma \left(\frac{\Omega_{s}\left({k^{\prime }}_{s}-{k^{\prime }}_{p}\right)+\Omega_{i}\left({k^{\prime }}_{i}-{k^{\prime }}_{p}\right)L}{2}\right)\right\}$$

where Ω*_s_* and Ω*_i_* are the frequency difference.

In addition to the phase-matching condition, we also consider the matching condition of group velocity [32]. But in the first SPDC, the derivative of the pump wave vector is always larger than that of the two down-converted photons. We use two Gaussian filters to remove the correlation of the two photons [33]. The two photons’ amplitude function becomes

(6) $$F(\omega_{s},\omega_{i})=T\left(\omega_{0}\right)T\left(\omega_{1}\right)\alpha \left(\omega_{s}+\omega_{i}\right)\varphi \left(\omega_{s},\omega_{i}\right)$$

where T(ωi)=exp(−Ωi2ςi2) is the corresponding filter. $\varsigma_{i}$ is the FWHM.

In the total cascaded process, the holistic Hamiltonian is the product of the Hamiltonian of two parts, that is *Ĥ* = *Ĥ*_1_*Ĥ*_2_. The expression of the last three photon states is

(7) $$|\psi_{3}\rangle =\int dt_{1}dt_{2}{\hat{H}}_{1}(t_{1}){\hat{H}}_{2}(t_{2})=B\int d\omega_{1}\int d\omega_{2}\int d\omega_{3}{\hat{a}}_{1}^{†}(\omega_{1}){\hat{a}}_{2}^{†}\left(\omega_{2}\right){\hat{a}}_{3}^{†}\left(\omega_{3}\right)F\left(\omega_{1},\omega_{2},\omega_{3}\right)|0\rangle +c.c.$$

When we determine the frequency distribution of the down-conversion of three photons, and there is no correlation between them, then the three photons amplitude can be equivalent to

(8) $$F\left(\omega_{1},\omega_{2},\omega_{3}\right)=\exp(-\Omega_{1}^{2}/\sigma_{1}^{2})\exp(-\Omega_{2}^{2}/\sigma_{2}^{2})\exp(-\Omega_{3}^{2}/\sigma_{3}^{2})$$

In practice, there are two sensitive parameters of the system that need to be strictly controlled: (1) the polarization stability of the light source and the optical path, and; (2) the temperature of the non-linear material. Both of them directly affect the refractive index of materials, thus affecting the phase-matching conditions.

### 2.3. Joint Spectrum and Purity

The simplest method to judge the frequency dependence of two photons produced by second order SPDC is to analyze their joint spectrum which is determined by

(9) $$JSI(\omega_{s},\omega_{i})={\left|F\left(\omega_{s},\omega_{i}\right)\right|}^{2}$$

The two photons are frequency uncorrelated if their joint spectrum is a circle or an ellipse parallel to the axis, which means the distribution of photons in frequency is independent of each other. It is impossible to obtain an optimal value simply by judging the shape or the angle with the coordinate axis because of the lack of a specific parameter to quantify the two-photon frequency correlation. Calculating the Schmidt number is the effective scheme to measure spectral correlation because it reflects the purity of correlation over frequency. It is defined as follows

(10) $$K=\frac{1}{\mathrm{Tr}\left\{\rho_{1}^{2}\right\}}=\frac{1}{P}$$

In this formula, $\rho_{1}$ is the density operator of photon *ω*_1_ and *P* represents the spectral purity. There is no frequency correlation between photon pairs when the Schmidt number K reaches the minimum value of 1. After calculating the Schmidt numbers with the parameters of crystal length and pump duration in each SPDC, the results are verified and analyzed by the joint spectrum of two photons under the optimum parameters. The joint spectral intensity of the photon triplets can be written as

(11) $$JSI(\omega_{1},\omega_{2},\omega_{3})={\left|F(\omega_{1},\omega_{2},\omega_{3})\right|}^{2}$$

We use the symbol quantity to carry on the maximum precision calculation. The result is converted to double type with 16 bits precision. The precision is enough that an ideal numerical simulation result can be obtained. Therefore, the error caused by the accuracy of software calculation can also be ignored.

## 3. Numerical Simulation Results

In this section, we discuss the generation of photon pairs from different materials, and finally obtain the pure-state photon triplets. Among the numerous nonlinear crystals, lithium niobite has a relatively higher nonlinear coefficient [34,35], which leads to a greater conversion efficiency. There is a wide range of transparency, from 420 nm to 5200 nm. In addition, lithium niobite doped with MgO has higher damage threshold, thus the periodically poled lithium niobate doped with 5% MgO (PPMgLN) will also be used as a reference for comparison. We get a set of crystal lengths which are optimum for each SPDC process through theoretical arithmetic.

In the first SPDC, the crystal and pump parameters are taken as the variables and the calculation of the Schmidt number is done. We select the appropriate pumping duration and crystal length *L*_1_ by analyzing the obtained data. After calculation, the frequency distribution of photonic *ω*_0_ is obtained. That is to say, the envelope information of the pump in the second SPDC is determined, which is $\exp[-(\omega_{0}-{\bar{\omega }}_{0})/\sigma_{0}^{2}]$, where *σ*_0_ is the bandwidth of the new source *ω*_0_. Then, the amplitude function of photon *ω*_2_ and *ω*_3_ is described as

(12) $$F\left(\Omega_{2},\Omega_{3}\right)=\exp\left[-\frac{{\left(\Omega_{2}+\Omega_{3}\right)}^{2}}{\sigma_{0}^{2}}\right]\exp\left(-i\frac{\Delta k_{2}L_{2}}{2}\right)\sin c\left[\frac{(k_{0}(\omega_{0})-k_{2}(\omega_{2})-k_{3}(\omega_{3})-k_{g2})L_{2}}{2}\right]$$

In the second SPDC, the Schmidt numbers of photon state between *ω*_2_ and *ω*_3_ are calculated by using the bandwidth information of the generated photons *ω*_0_ and taking the crystal length *L*_2_ as the variable. Then we select the appropriate crystal length *L*_2_. Each time the most appropriate parameters are determined, the two-photon joint spectrum and the final three-photon joint spectrum are given to verify the theoretical calculation.

### 3.1. Realization of Pure-State Photon Triplets in PPLN

**Figure 2:**
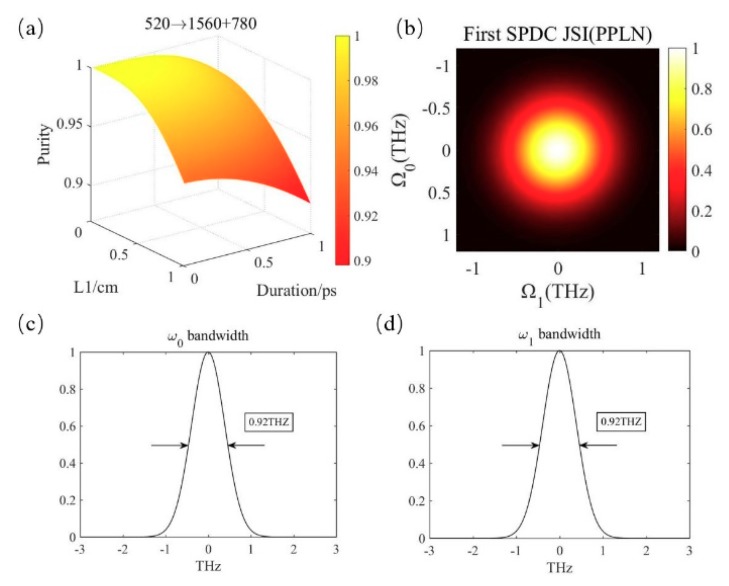
(**a**) The spectral purity in the first SPDC using PPLN. (**b**) The joint spectrum of photon 1 and 0. (**c**,**d**) The bandwidth of the photon pairs.

The pump wavelength is 520 nm. Relevant data in the first down-conversion is shown in Figure 2. The z axis in Figure 2a describes the variation of the spectral purity with the parameters. The x-axis represents the range of the selected crystal lengths from 0 to 1 cm while the y-axis is the variation of pump duration in the range of 0–1 ps. The wavelengths of the pair of entangled photons are *λ*_1_ = 1560 nm and *λ*_0_ = 780 nm. The periodicity of the first PPLN is 38.47 μm. Due to the Gaussian filter with a bandwidth of 0.8 THz, the spectrum purity between photons *ω*_0_ and *ω*_1_ is almost 1 in the region where the crystal length and pump duration are smaller. Considering the realizability, we selected a pump duration of 100 fs and a crystal length of 0.2 cm.

Figure 2b describes the joint spectral intensity of photons *ω*_1_ and *ω*_0_. It is intuitive to see that there is no frequency correlation between the two photons. Figure 2c,d are the bandwidth of photons *ω*_1_ and *ω*_0_, respectively. Because the transmission of the filter is related to the bandwidth, the down-conversion photons of the two channels have the identical frequency distribution.

**Figure 3:**
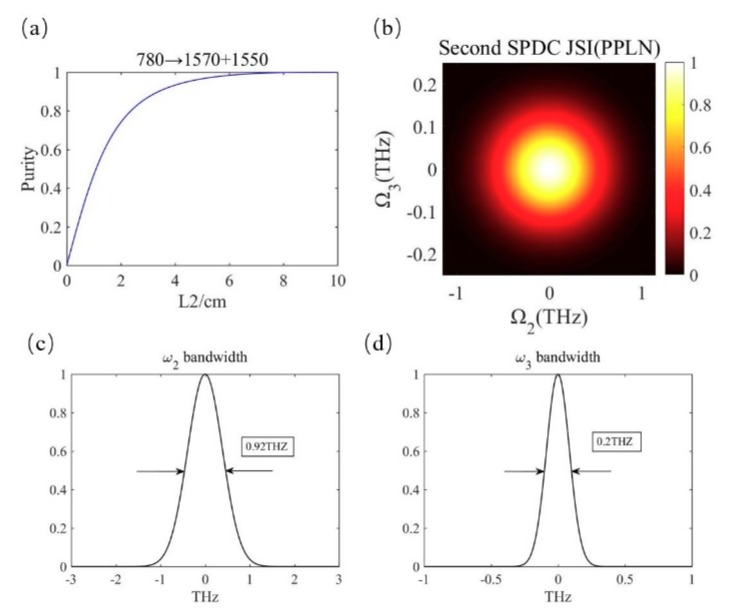
(**a**) Spectral purity data in the second SPDC. (**b**) The joint spectrum of photon 2 and 3. (**c**,**d**) The bandwidth of the photon pairs.

In the second SPDC process, we select the generation wavelengths of *λ*_2_ = 1570 nm and *λ*_3_ = 1550 nm in consideration of the matching condition of the group velocity. The polarization period of the second PPLN is 88.76 μm. Relevant data are shown in Figure 3. Figure 3a describes the calculation of the spectral purity of photons *ω*_2_ and *ω*_3_ with the crystal length *L*_2_ as the independent variable. It can be seen that with the increase of crystal length, the purity increases to the maximum value of 1. We chose the best crystal length *L*_2_ as 9.16 cm. Figure 3b is the joint spectral intensity of photon pair *ω*_2_ and *ω*_3_. It can be seen that the photon pairs are still frequency uncorrelated in the second SPDC process. As shown in Figure 3c,d, the bandwidth of photons *ω*_2_ and *ω*_3_ is different because the perfect group velocity match is not achieved, but this does not affect the correlation between them.

**Figure 4:**
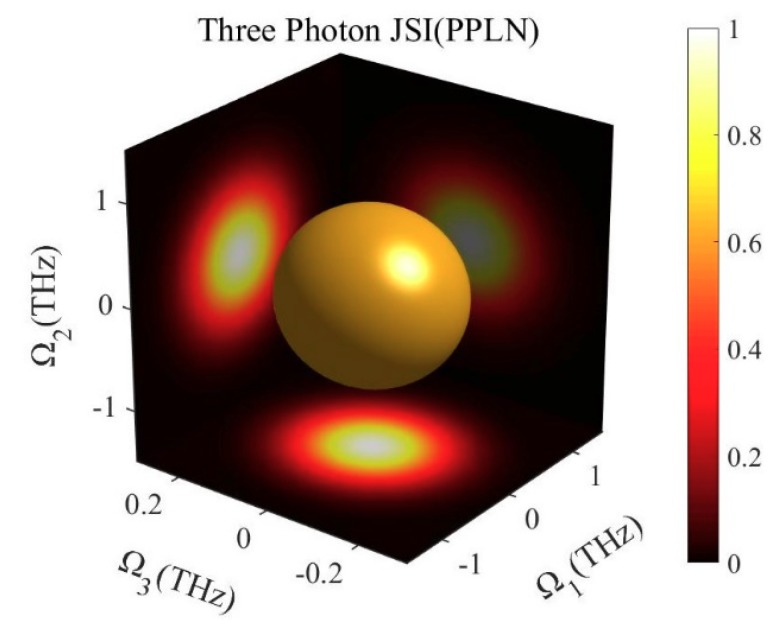
Joint spectrum of the three photons generated from the cascaded PPLN.

Since photons *ω*_1_ and *ω*_0_ are not correlated in frequency, both photon (1,2) and photon (1,3) should be irrelevant theoretically. Figure 4 shows the joint spectrum of photon triplets. From the relationship between each two-photon, as shown in three projection planes, there is no correlation between photons *ω*_1_ and *ω*_2_, *ω*_1_ and *ω*_3_. So far, we have obtained photon triplets which are not related in the frequency dimension. At the same time, all three of them are in the C-band.

### 3.2. Realization of Pure-State Photon Triplets in PPMgLN

We also chose 520 nm as the pump wavelength for comparison. The group velocity matching condition of the second SPDC is not satisfied. The wavelength of photon ω_1_ and ω_0_ generated in the first down-conversion are 1520 nm and 790.4 nm, respectively.

**Figure 5:**
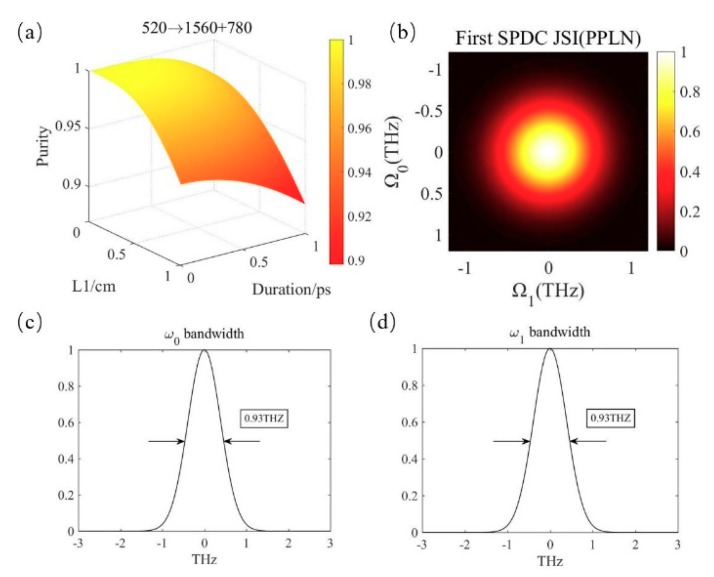
(**a**) The spectral purity in the first SPDC using MgO-doped PPLN. (**b**) The joint spectrum of photon 1 and 0. (**c**,**d**) The bandwidth of the photon pairs.

**Figure 6:**
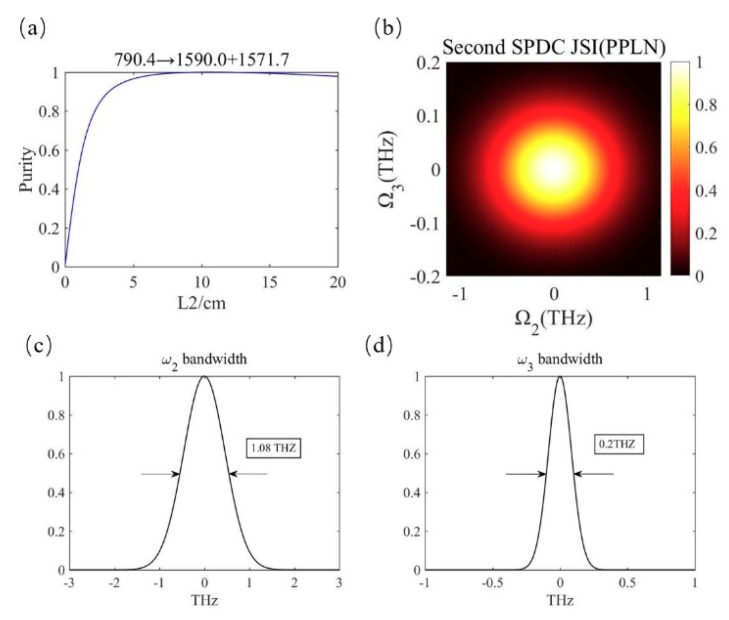
(**a**) The spectral purity data in the second SPDC. (**b**) The joint spectrum of photon 2 and 3. (**c**,**d**) The bandwidth of the photon pairs.

The results of correlated data are given in Figure 5. The photon wavelengths generated by the second down-conversion are *λ*_2_ = 1590 nm and *λ*_3_ = 1571.7 nm, pumped by photon *ω*_0_. Similar data are shown in Figure 6. The pump duration is 0.27 ps while the crystal lengths are *L*_1_ = 0.2 cm and *L*_2_ = 10.74 cm (corresponding Ʌ_1_ = 34.85 μm and Ʌ_2_ = 83.51 μm).

**Figure 7:**
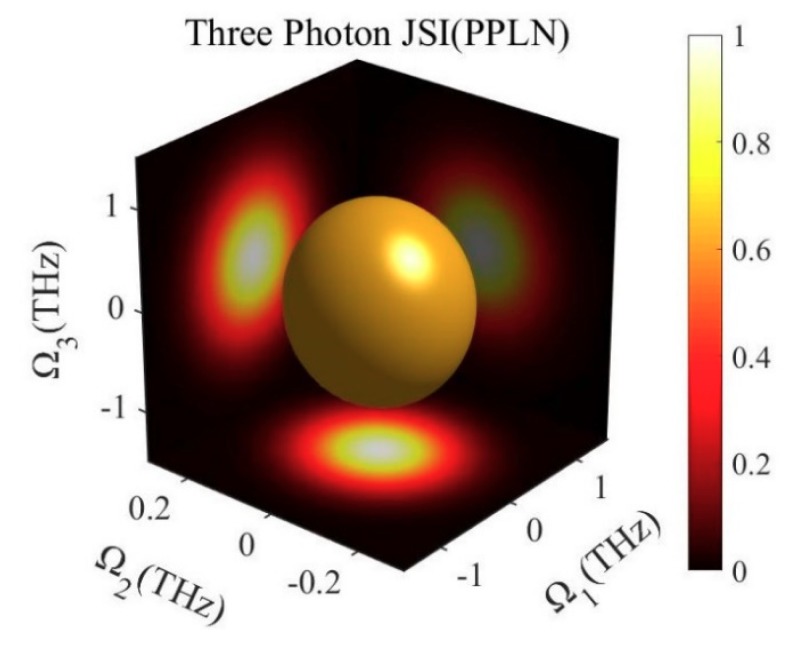
Joint spectrum of the three photons generated from the cascaded SPDC MgO-doped PPLN.

We also produce three photons with a purity of 100%. Due to the material differences, the center wavelengths of the photons *ω*_2_ and *ω*_3_ are longer than in PPLN. It takes a slightly longer crystal than PPLN to achieve the phase-matching condition. The bandwidth of the photons *ω*_1_ and *ω*_2_ generated in the PPMgLN is relatively wider. Figure 7 shows the joint spectral intensity of photon triplets generated by cascaded PPMgLN. The three projection planes reflect the correlation between two of the three photons.

PPLN is more suitable for weak light due to the better phase-matching conditions. According to our theoretical results, the wavelength distribution of the three-photon generated in PPLN is closer. For the same pump (*λ_p_* = 520 nm), three photons with wavelengths of 1550 nm, 1560 nm and 1570 nm can be realized in PPLN. PPMgLN is more suitable for the pump with higher intensity, because doping MgO can increase the damage threshold of the material and obtain higher brightness photon triplets. But we can only obtain photons with wavelengths of 1520 nm, 1590 nm and 1571.7 nm. In the preparation of the light source, spectral purity is one of the core indicators. The purpose of our work is to prepare photon triplets of spectral pure-state (frequency uncorrelated), which provides a reliable scheme for the preparation of high quality sources in the field of quantum technology. There is no prior research on pure state photon triplets in the C-band before our work. Our method can also provide a heralding pure-state biphoton source with higher interference visibility. Compared with the unpredicted conditions, the heralding two-photons have superior advantages, such as avoiding the detection of noise photons, which greatly reduces the bit error rate (BER). It guarantees the realization of many of these tasks relying on qubits that are encoded in the polarization states of single photons.

## 4. Conclusions

In summary, we discuss how to select the optimal pump and crystal parameters to obtain the pure state photon triplets by cascaded second-order SPDC using PPLN and MgO-doped PPLN. We chose 520 nm as the pump source with duration of 0.1 ps. After calculating the Schmidt number of photon pairs generated in each SPDC, we determined the crystal lengths of the two PPLN are 0.2 cm and 9.16 cm, respectively. The lengths of the two PPMgLN are 0.2 cm and 10.74 cm, respectively. According to theoretical calculation, the purity of photon pairs from each SPDC can reach 100%, that is, there is no frequency correlation. We have achieved photon triplets with a spectral purity of 100% in the C-band. We firmly believe that in the future development of quantum networks, our scheme can provide reliable pure-state photon triplet sources for various quantum information processes.
